# Obesity as a Risk Factor for Dementia and Alzheimer’s Disease: The Role of Leptin

**DOI:** 10.3390/ijms23095202

**Published:** 2022-05-06

**Authors:** Juan Antonio Flores-Cordero, Antonio Pérez-Pérez, Carlos Jiménez-Cortegana, Gonzalo Alba, Alfonso Flores-Barragán, Víctor Sánchez-Margalet

**Affiliations:** Department of Medical Biochemistry and Molecular Biology and Immunology, Medical School, Virgen Macarena University Hospital, University of Seville, Av. Sánchez Pizjuan 4, 41009 Sevilla, Spain; jaflores@us.es (J.A.F.-C.); antonioresi@gmail.com (A.P.-P.); cjcortegana@us.es (C.J.-C.); galbaj@us.es (G.A.); alfonreyes1992@gmail.com (A.F.-B.)

**Keywords:** obesity, leptin, inflammation, leptin resistance, Alzheimer’s disease

## Abstract

Obesity is a growing worldwide health problem, affecting many people due to excessive saturated fat consumption, lack of exercise, or a sedentary lifestyle. Leptin is an adipokine secreted by adipose tissue that increases in obesity and has central actions not only at the hypothalamic level but also in other regions and nuclei of the central nervous system (CNS) such as the cerebral cortex and hippocampus. These regions express the long form of leptin receptor LepRb, which is the unique leptin receptor capable of transmitting complete leptin signaling, and are the first regions to be affected by chronic neurocognitive deficits, such as mild cognitive impairment (MCI) and Alzheimer’s Disease (AD). In this review, we discuss different leptin resistance mechanisms that could be implicated in increasing the risk of developing AD, as leptin resistance is frequently associated with obesity, which is a chronic low-grade inflammatory state, and obesity is considered a risk factor for AD. Key players of leptin resistance are SOCS3, PTP1B, and TCPTP whose signalling is related to inflammation and could be worsened in AD. However, some data are controversial, and it is necessary to further investigate the underlying mechanisms of the AD-causing pathological processes and how altered leptin signalling affects such processes.

## 1. Introduction

When focusing on dementia, it is worth noting that the separating line between normal and pathological aging is not adequately defined. Therefore, it is difficult to determine where each phenomenon begins and ends and to distinguish the common deficiencies and individual differences within these phenomena [1]. This narrow line that separates the normal from the pathological is where we must intervene to minimise the symptoms the disease produces, making an early diagnosis of dementia necessary. The difficulty is even greater taking into account that the aging process itself can produce negative effects on general health and cognitive function in particular. Similarly, aging is linked to an increase in body weight, adiposity, and variations in hormones and adipokines, showing an altered pattern with age [2,3]. Similarly, both in murine and human models, an increase in microglial reactivity and inflammation with age has been described [2,3]. In this review, we discuss how this altered pattern is a factor that predisposes to obesity and dementia, such as Alzheimer’s disease (AD).

Procedures have been developed over time to identify patients with early onset dementia, a concept that has evolved into the current term, mild cognitive impairment (MCI). Ronald Petersen developed the concept of MCI through the Mayo Clinic. The concept was an improvement in its attempt to identify people who may progress to dementia, as the cognitive aspects were introduced to the pre-existing aspects of memory. Currently, the fundamental objective of dementia research is to find markers that provide an early diagnosis and thus enable action to be taken before the disease evolves [1]. MCI is an example of this.

Today, obesity is growing in the global population due to multiple causes: lifestyle, stress, nutrition, genetic background, and lack of exercise. In obesity, white adipose tissue (WAT) not only stores excess energy but also disturbs endocrine function. WAT secretes a group of substances called adipokines that exert autocrine, paracrine, and endocrine effects at the systemic level and also centrally in the central nervous system (CNS) [4,5,6]. Obesity has been linked to cognitive deficits, impaired long-term potentiation and synaptic plasticity, and a smaller brain volume, increasing the probability of developing Alzheimer’s disease (AD) and other dementias [7]. Thus, obesity is established as a risk factor for dementia. Furthermore, obesity causes a state of low-grade chronic inflammation in adipose tissue that leads to the dysregulation of homeostatic systems, which in turn leads to the development of various diseases, including those related to neurodegeneration. During this process, adipose tissue produces an increase in pro-inflammatory adipokine levels (interleukin 1 beta (IL-1β), interleukin 6 (IL-6), and tumoural necrosis factor alfa (TNF-α), and leptin) and a decrease in anti-inflammatory adipokine levels, such as adiponectin [4,6]. Yet another essential component of these complex interrelationships between obesity and brain status is the gut microbiota. In fact, meticulously detailed in a review by authors [8], it is explained how an altered intestinal microbiota pattern (or dysbiosis) can lead to a permanently altered physiological pattern, which can lead to cognitive impairments due to alterations in the gut-brain axis. In addition, it indicated that the administration of pre- and probiotics can restore this dysbiosis, enabling a return to an adequate homeostatic balance.

Leptin, a pro-inflammatory adipokine secreted by WAT and found to be increased in people with a high body mass index, acts centrally at the level of the hypothalamic region through anorexigenic proopiomelanocortin (POMC)/cocaine- and amphetamine-regulated transcript (CART) neurons and orexigenic neuropeptide Y (NPY)/agouti-related peptide (AgRP) neurons, controlling food intake and energy expenditure. The literature suggests that leptin may be the link between obesity and dementia through the development of inflammation. Therefore, we will analyse the information on inflammation-induced leptin resistance that occurs during obesity as a pathological mechanism that may underlie neurodegenerative diseases such as AD and other dementias.

## 2. Obesity and Dementia

Obese individuals are at greater risk of developing age-related cognitive decline, vascular dementia, MCI, and AD [4], as well as other neurodegenerative pathologies such as Parkinson’s [9,10] and Huntington’s disease [10,11]. In this section, we give an overview of obesity as a risk factor for dementia based on adipose tissue measurement indices, brain structural changes, and cognitive impairment measurements. Our purpose is to identify modifiable risk factors that allow an early diagnosis and treatment.

Obesity can be defined as an excessive accumulation of adipose tissue which generates a low-grade inflammation state, whereas AD can be defined as a progressive neurodegenerative disease whose distinctive histopathological characteristics are the extracellular amyloid plaques and intracellular neurofibrillary tangles.

A parameter used as a measure of adiposity is the body mass index (BMI). However, its use has not been free from difficulties. That is why the waist circumference and the waist-to-hip ratio (WHR) have also been used to evaluate excess fat [12]. In fact, in the work by Beyer et al. (2019) [12], it is suggested that the WHR, as part of a metabolic obesity profile, is a determining factor that plays a role in grey matter volume reductions, which might lead to reduced cognitive functions, that have a weaker association when using the BMI [12,13]. Nevertheless, BMI is the most widely used adiposity index [14,15,16]. Thus, an association between BMI and dementia has been described, although this relation is controversial [14,15,16,17]. Firstly, it has been suggested that being overweight and obese in middle age is related to a higher risk of dementia in old age. Nevertheless, a high BMI in late life is associated with better cognition [6,16,17]. Secondly, other studies have described contradictory outcomes, where a lower risk of dementia was observed for very obese people (BMI > 40 kg/m2) while underweight people (BMI < 20 kg/m2) display a higher dementia risk than heavyweight people [18]. There are different epidemiological studies that relate obesity and dementias, such as the more frequent AD, but, in general, these studies find a positive correlation between obesity and cognitive impairment, with a possible U-shaped curve. In this context, a reverse relation has been described between obesity and grey matter and whole brain volume [19,20,21,22,23,24,25], even though a minor number of publications did not find such a relation [26,27].

As mentioned above, obesity generates a chronic low-grade inflammation state, which is characteristic of a variety of other chronic conditions, such as metabolic syndrome, non-alcoholic fatty liver disease, type 2 diabetes mellitus, and cardiovascular disease [28,29], as well as neuroinflammation [30,31,32], a hallmark of neurodegenerative diseases such as AD [33,34,35]. Following this line, different animal studies have confirmed the connection between obesity and cognitive mismatches and/or impairment. Thus, different works point to an altered cognitive function when administering a less-healthy diet. A high-fat diet (HFD) rich in saturated fatty acids can result in obesity as well as deficits in hippocampal-dependent learning and memory functions [36,37]. Male Wistar rats fed with a HFD showed impaired memory, an effect that was augmented with a longer duration of HFD consumption [38] while, similarly, rats fed with a high-fructose-high-coconut oil diet experienced impaired hippocampal-dependent learning and memory processes, as evaluated through the Morris water maze task [39]. In another study [40], HFD-induced brain insulin resistance and cognitive impairment were observed. Molecular changes, such as a significant decrease in tyrosine phosphorylation of the insulin receptor and increased serine phosphorylation of IRS-1, which are signs of insulin resistance, might be the cause of the cognitive impairment in this mouse model. These molecular changes were accompanied by inflammatory signalling (NFκB, JNK) and stress responses (p38 MAPK, CHOP) in whole brain lysate. In a transgenic rat model of pre-AD and MCI, impaired special learning and memory has been described in the Morris water maze when rats received a high-caloric diet [41]; at the same time, some parameters of brain inflammation, such as microgliosis, were also found. Moreover, activated OX-6+ microglia were detected, as well as GFAP+ astrocytes located predominantly in the white matter, and the synaptic density in the CA1 and CA3 hippocampal subregions was lower in this high-calorific diet. In a triple transgenic AD mice model (3xTg-AD), an impairment in the cognitive function has been shown when administering a HFD, and this diet was able to induce enhanced oxidative stress and aggravated neuronal apoptosis via inactivation of the Nrf2 signalling pathway [42]. So, numerous animal studies indicate the relation between obesity and AD and other forms of dementia that affect cognitive function.

Overall, obesity seems to be a risk factor for different forms of dementia, where we can find long-term memory and attention impairment, and executive function deficits. We have reviewed how cerebral structural and functional changes in obese people occur, and how a high saturated fat diet can affect cognitive function, including brain inflammation as a hallmark of this process.

## 3. Obesity and Leptin

Energy homeostasis and the maintenance of body weight require that the peptides secreted by the organs of the periphery establish a signalling and functional interrelation between them. To do this, adipocytes synthesise and secrete adipokines with pleiotropic effects in various tissues and regulate numerous physiological functions [43,44]. In this review, we will focus on leptin, due to its involvement in the regulation of food intake and energy expenditure, studying its involvement in dementia and its relationship with obesity.

Leptin has been found to circulate in plasma in proportion to body fat mass and this can be taken as an indicator of adiposity [45,46]. However, its study is difficult, since its levels vary by multiple factors: sex, BMI, starvation, and energy states. In addition, it shows a circadian rhythm, with leptin levels at their maximum between midnight and dawn, and is affected by other hormones and cytokines [47]. Leptin is a 16 kilodalton (kDa) peptide hormone produced by the ob (obese) gene. Leptin receptors are class I cytokine receptors. Previous studies have described six isoforms of the mRNA variants of the gene encoding the leptin receptor: four short forms (LepRa, LepRc, LepRd, LepRf), one long form (LepRb), and an extracellularly secreted soluble form (LepRe) [48]. Furthermore, five of the six isoforms (LepRa-LepRd and LepRf) have common extracellular and transmembrane domains, and only the soluble form (LepRe) has only an extracellular domain [48]. The long form LepRb has intracellular domains and proline-rich regions known as box1, box2, and box3 that are associated with Janus kinase (JAK) and signal transducer activators of transcription (STAT) signalling activation, whereas the other isoforms seem to be implicated in the leptin transport from the periphery to the CNS through the blood-brain-barrier (BBB) [48,49,50,51,52]. Leptin receptors are expressed throughout the body. In the CNS, leptin receptors are expressed in the arcuate nucleus (ARC), the dorsomedial hypothalamus (DMH), the ventromedial hypothalamus (VMH), the lateral hypothalamus (LH), the mediobasal hypothalamus (MBH), and the paraventricular nucleus (PVN). In addition, they have also been found in the cerebral cortex, hippocampus, ventral tegmental area, substantia nigra, medulla, and cerebellum [48,50,52,53,54,55,56,57]. Furthermore, the short isoforms LepRa and LepRc are expressed in the brain microvessels that make up the BBB, suggesting that these receptors may be associated with leptin transport [53,54]; in fact, the LepRa and LepRc isoforms have been shown to mediate leptin transport in the BBB, and dysfunctional receptors can lead to leptin resistance [56]. The expression of the leptin receptor has been observed in different neurons, including glutamatergic, GABAergic, and dopaminergic neurons. Thus, there is evidence of its involvement in the mechanisms of long-term potentiation, long-term depression, and motivational eating [42,44,45,48,49].

Biochemically, when leptin binds to its receptor it allows the activation of Janus kinase 2 (JAK2) and its autophosphorylation. Once activated, JAK2 phosphorylates other tyrosine residues (Tyr) in the receptor, including Tyr985, Tyr1077, and Tyr1138, to mediate different intracellular signalling pathways [48,58,59]. Intracellular signalling pathways initiated by the leptin receptor are [48,53,58,60,61]:

1. JAK/signal transducer activators of transcription 3 (STAT3);
2. Phosphatidylinositol 3-kinase (PI3K)/protein kinase B (Akt);
3. Extracellular signalling-regulated kinases (ERK); and
4. Signal transducer activators of transcription 5 (STAT5).

As we will see below, leptin is involved in various aspects of cognition, neurogenesis, neuroprotection, synaptic plasticity, and structural changes [57,62], which is not surprising given its access from the periphery to the CNS, the number of leptin receptors, and its wide distribution in the brain.

## 4. Leptin; Its Relationship with Cognition and Synaptic Function

An important aspect of dementia is cognitive deterioration, which is manifested in different aspects such as synaptic function, learning, and memory. So, before addressing the connection between leptin and dementia, it is important to analyse the relationship and implication of leptin with these different aspects of cognition. Leptin signalling regulates different processes including food intake, energy expenditure, cognition, learning, memory, and mood [63]. Studies show that leptin performs different neuroprotective functions [56,57,63,64,65]. Furthermore, leptin regulates neurogenesis, synaptogenesis, neuronal excitability, and neuroprotection [57,62].

In learning and memory processes, synaptic plasticity, and the relationships between neurons at the level of synaptic transmission, are fundamental. Furthermore, in these processes, the mechanisms of long-term potentiation (LTP) and long-term depression (LTD) of synaptic transmission are crucial for the formation and consolidation of hippocampal memory. Thus, leptin is involved in these processes at the level of the hippocampus where leptin and glutamate receptors {N-methyl D-aspartate (NMDA) and α-amino-3-hydroxy-5-methyl-4-isoxazolepropionic acid (AMPA) receptors} are key players [4,50,59]. In support of this evidence, leptin receptors have been found in neuronal and non-neuronal cells in the hypothalamus, the cerebral cortex, as well as in the dentate gyrus, and the CA1 and CA3 areas of the hippocampus [62]. This last finding is fundamental, as these areas are highly involved with cognition and memory. Recent studies indicate that NMDA receptors are necessarily involved in leptin-dependent homeostatic control of body weight [66]. Thus, leptin enhances NMDA receptor function by promoting the efficiency of excitatory synaptic transmission at hippocampal Schaffer-collateral (SC)-CA1 synapses and allowing the conversion of short-term potentiation into LTP. Therefore, it is not surprising to have found LTP and LTD deficiencies in leptin-insensitive obese mice (db/db) and rats deficient in the leptin receptor (fa/fa) [4,59]. Consequently, leptin improves hippocampal-dependent memory. Thus, in the review by McGregor and Harvey [66] it is found that in transgenic mouse models of Alzheimer’s disease (CRND8), leptin improved performance in object recognition, contextual, and fear-conditioned tasks. In addition, performance in hippocampal-dependent memory tasks was also optimised in mice prone to accelerated senescence (SAMP8) that have elevated levels of the β-amyloid peptide (Aβ).

Leptin can also regulate the configuration of neurons. Thus, plasma leptin levels have been associated with grey matter volume in various brain regions. Exogenous leptin administration recovers structural brain abnormalities in congenitally leptin-deficient humans and reverses the abnormalities in ob/ob mice [62]. Interestingly, the study by Annweiler [64] finds a relationship between circulating leptin levels and U-shaped cognition. Their results suggest that older adults with low or high circulating leptin concentrations are particularly prone to cognitive impairment and point to leptin as a modifiable risk factor.

Therefore, we can say that leptin, through its actions in the periphery or the CNS, produces neuroprotective effects that improve cognitive function and memory. As a result, its dysfunction due to a deficit in signalling pathways, a decrease or alteration of its levels, or leptin resistance can cause processes that promote neurodegeneration [62].

## 5. Leptin Resistance: Mechanisms Involved

Leptin is reduced during fasting and increases with food consumption. When we eat, adipocytes release leptin, which tends to reduce eating behaviour. However, the paradox exists that in obesity, the leptin levels are increased; despite this, there is no indication or signal of satiety due to a peripheral and/or central resistance to leptin. Thus, an imbalance between anorexigenic and orexigenic signals occurs, which leads us to receive a signal of decreased satiety, and contributes to obesity, leading to a vicious cycle [4,61].

Resistance to leptin indicates that despite high plasma levels in obese people, signalling in the hypothalamus is decreased [4]. The foregoing leads us to review and analyse the factors involved in this dysfunction in leptin signalling. Next, we analyse the mechanisms that have been proposed as an explanation for leptin resistance, which is related to obesity, and helps to develop or perpetuate diseases such as dementia: (a) genetic mutations, (b) alterations in the transport of leptin through the BBB, (c) desensitisation of leptin receptors that reduce the functionally active number, (d) attenuation of signalling leptin {suppressor of cytokine signalling type 3 (SOCS3), protein tyrosine phosphatase type 1B (PTP-1B), and protein tyrosine phosphatase of T cells (TCPTP)}, (e) alteration of cellular descending leptin signalling in the CNS, and (f) multiple factors such as inflammation, elevated levels of C-reactive protein (CRP), adapter protein1 (SH2B1), and endoplasmic reticulum (ER) stress as well as decreased activity of histone deacetylase and the type of diet [58,61,67,68,69,70]. In this context, obesity-related inflammation may contribute to leptin resistance, which in turn further increases weight gain [71] and leptin levels, which then increases the inflammation-related obesity, since leptin is an important proinflammatory adipokine [72,73,74], activating monocytes [75] and lymphocytes [76].

### 5.1. Pathways of Leptin Entry into the Brain and Leptin Resistance

There are several pathways for the entry of leptin into the brain: through the (a) endothelial cells of the BBB, (b) epithelial cells of the choroid plexus, and (c) through the medio basal hypothalamus, which is surrounded by tanicytes that form a barrier between the median eminence and the cerebrospinal fluid [77,78,79]. Existing evidence shows that leptin traverses the BBB through a saturable transport system, but there are doubts about the involvement of LepRa [51,52,61,77]. An investigation has found that brain endothelial cells express higher levels of LepRa than LepRb or megalin receptor 2 (Lrp2). Furthermore, leptin has a higher affinity for both LepRa and LepRb, suggesting that LepRs are the most important binding sites for leptin, rather than Lrp2 [79]. Likewise, in another report, it has been suggested that the leptin receptor is necessary in the brain barrier for the uptake of leptin by the brain and the regulation of the food reward; megalin receptor 1 (Lrp1) and Lrp2 are considered alternative transporters [77]. However, the work by Bartolome et al. [80] provides evidence against the leptin receptor. Here, it has been suggested that LepR is not the main leptin transporter in human BBB, but rather the megalin receptor, since the authors observed a reduction in brain leptin uptake by eliminating the megalin receptor in the endothelial cells of the BBB, which induced hyperphagia and obesity.

In the literature, there are two contradictory currents of thought as to whether the leptin resistance at the level of the BBB is due to a deficient leptin transport. On the one hand, there are studies that support the idea that there is a decrease in leptin transport through the BBB. In this sense, circulating factors in the blood, such as triglycerides, could reversibly inhibit leptin transport. Thus, low levels of these factors allow the transport of leptin through the BBB, but high triglyceride doses can inhibit it. Therefore, treating triglyceride levels in the blood could decrease central (and peripheral) resistance to leptin and reduce obesity and the cognitive problems associated with it [4,78,81]. This idea is also supported by Di Spiezio [77] who states that a deficit in the transport of leptin to the brain can increase the food reward. Furthermore, obese rodents have been shown to respond to the central administration of leptin through intracerebroventricular injections, but not to peripheral administration of leptin through intraperitoneal injections, which may be indicative of a possible impairment in transport through the BBB [68,81]. On the other hand, it has also been hypothesised that there is no deficit in the transport of leptin through the BBB. In this sense, it has been found that obese mice, as well as lean mice, retain a functional leptin transport system without leptin deficiency in the circumventricular organs, with the choroid plexus and the mediobasal hypothalamus playing a key role [67]. Another finding that supports the idea that there is no deficient transport of leptin through the BBB is that of the Bardet-Biedl Syndrome (BBS) mouse model, characterised by retinal dystrophy, polydactyly, renal and gonadal anomalies, cognitive impairment, and obesity, and, where proteins of an octameric complex, the “BBSome”, are mutated, leading to impaired transport of the leptin receptor LepRb to the plasma membrane in hypothalamic cells, thereby preventing intracellular signalling which causes resistance to leptin and obesity [82]. 

These contradictory results warrant further investigation in order to clarify the role of leptin transport through the BBB in leptin resistance. 

### 5.2. Inflammation and Leptin Resistance

It has been suggested that leptin resistance in obesity might start through the activation of inflammatory signalling [69]. It is generally accepted that, in obesity, hypertrophic adipocytes suffer an increase in proinflammatory cytokines expression and secretion that have periphery and brain effects [83,84]. Furthermore, several adipokines, and interleukins, in particular, are associated with the inflammatory processes implicated in dementia [6,83]. The production of interleukin 6 (IL-6), which is triggered by a lack of exercise and by saturated fat-rich diets, can lead to resistance to anorexigenic signals in the hypothalamus [52].

Low-grade inflammation due to obesity drives human C-reactive protein (CRP) production by hepatocytes in vitro and in vivo in humans [85]. It has been found that peripheral human CRP can reduce the amount of human leptin that enters the CNS, preventing its transport across the BBB and into the median eminence. Furthermore, once inside the CNS, it reduces the physiological function of human leptin [85]. So, both mechanisms may contribute to leptin resistance. Therefore, we can consider CRP a mechanism involved in inflammation-mediated leptin resistance.

Inflammation causes central resistance to leptin through the expression of SOCS3. Thus, it has been found that SOCS3-stimulated adipocyte apoptosis worsens inflammation and inhibits the activity of the JAK2/STAT3 signalling pathway [86].

Many advances have been made through studies seeking to ameliorate obesity and hyperphagia produced by diets high in fatty acids. Interestingly, positive results in these studies have involved reduced inflammation and/or increased leptin sensitivity. As evidence of the above, it has been found that the peripheral delivery of the interleukin 10 (IL-10) gene using an adeno-associated virus (AAV) is capable of suppressing inflammation in the hypothalamus arcuate nucleus (ARC) in mice with diet-induced obesity (DIO) and decreasing hyperphagia and obesity [87]. Another study finds that proinflammatory cytokines in the brain increased Toll-like receptor 4 (TLR4) expression, ER stress, and nuclear factor kappa light chain enhancer of activated B cells (NF-kB) expression. Furthermore, this led to increased inflammation and leptin resistance, which was reduced by treatment with pyrogallol-phloroglucinol-6,6-bieckol (PPB) [84]. The central administration of docosahexaenoic acid (DHA, 22:6 n-3) reduced high-fat diet (HFD)-induced inflammation and obtained beneficial effects by inhibiting SOCS3 [88]. It has been found that the use of the plant terpenoid compound ginsenoside Rb1 reduces body weight gain, the accumulation of fat mass, and the expression of inflammatory markers, SOCS3 and PTP1B, in addition to reducing the deterioration of the signalling pathway JAK2-STAT3 and central leptin sensitivity due to obesity induced by high fatty acid diets [89,90].

Taken together, these studies suggest that neuroinflammation could cause leptin resistance, so acting on inflammation may be a way to reduce leptin resistance. In support of this, it is worth noting that a study related to inflammation and the signalling pathway of NF-KB mediated by sirtuins (SIRT) shows that its regulation can play an important role in neuroinflammation, as well as in local inflammation [91]. In addition, the NF-kB factor appears to play an important role in hypothalamic leptin resistance [92].

In summary, the data suggest that a HFD may reduce leptin sensitivity and that obesity may induce inflammatory processes. Furthermore, the studies and the scientific evidence presented seem to indicate that reducing inflammation can act on the pathways involved in leptin resistance, producing an improvement in leptin or its sensitivity [92] and obtaining favourable results on obesity. Likewise, SOCS-3 is hinted at as a mechanism mediating leptin resistance.

### 5.3. Hypothalamic Endoplasmic Reticulum Stress and Leptin Resistance

Endoplasmic reticulum (ER) stress is currently being implicated as a mechanism that can lead to leptin resistance [93]. Thus, early exposure to stress has been found to alter factors in the adipose tissue and leptin system that led to increased body fat accumulation when exposed to a western diet later in life [5]. An aldehyde, 4-hydroxy-2-nonenal (4-HNE), may be involved in the development of leptin resistance in neuronal cells. Levels of this aldehyde can rise with fat accumulation which can initiate an ER stress response and further increase its production, and can then lead to impaired leptin signalling and obesity [94]. The elevated free fatty acid concentration in the hypothalamus can lead to lipotoxicity. Furthermore, a relationship between hypothalamic lipotoxicity and ER stress has been suggested as a possible explanation for the onset of obesity [95].

Alternatively, various compounds with the ability to decrease ER stress and/or ER stress-induced leptin resistance have been reported [93]. Notably, fluvoxamine has the ability to reduce ER stress through the sigma-1 receptor (Sig-1R) and its effect on reducing leptin resistance may be due to this mechanism. Celastrol, a chemical chaperone, ameliorates hypothalamic ER stress and obesity in mouse models. Chemical chaperones are compounds that have been suggested to diminish ER stress-induced leptin resistance. Flurbiprofen has also been found to improve ER stress-induced leptin resistance and HFD-induced obesity. Collectively, the above information allows us to affirm that certain compounds favour ER stress and increase leptin resistance while others decrease it and improve leptin resistance [93]. Therefore, future research should focus on investigating the pathways through which these compounds carry out their beneficial or detrimental effects.

Lipotoxicity has been linked to ER stress and the downstream melanocortin system, in that the suppression of mitofusin2 (Mfn2) in proopiomelanocortin (POMC) neurons causes stress in the ER, thus altering the processing of melanocyte-stimulating hormone (α-MSH) and causing leptin resistance and obesity. Therefore, administration of the chemical chaperone 4-phenylbutyrate (4-PBA) was able to correct the tendency of the endogenous melanocortin 4 receptor (MC4R) to fold incorrectly and restore the correct response of α-MSH [95].

Sleep fragmentation has also been linked to hypothalamic ER stress. Specifically, the former induces the latter, leading to hyperphagia and leptin resistance. What is significant in this study is the observation of an increase in PTP1B expression and activity due to sleep fragmentation. The authors conclude that while sleep fragmentation seems to induce leptin resistance through PTP1B, high-calorie diets would induce it through SOCS3. However, this situation is reversed with a chemical chaperone treatment or with transgenic ablation of CHOP-/+ [96]. Therefore, the PTP1B and SOCS3 negative feedback pathways seem to affect leptin resistance; specifically, the PTP1B pathway would be affected by hypothalamic ER stress and the SOCS3 pathway would be affected by high-calorie diets.

Finally, we can point out that ER stress induces leptin resistance. Furthermore, the POMC hypothalamic neurons of the arcuate nucleus are vulnerable to ER stress [95] and leptin exerts its effects through them. Therefore, we accept the implication of ER stress in leptin resistance, but it is possible that this is due to the interaction or combination of some of the different mechanisms which induce it.

### 5.4. Reduced Sensitivity to Leptin (Receptors, Receptor Downstream Signaling, and Negative Feedback Signaling Pathways)

Certain intracellular factors such as SOCS3, PTP1B, and TCPTP negatively control leptin signalling and may be involved in leptin resistance [92]. Thus, the elimination or attenuation of SOCS3 expression produces beneficial effects on leptin sensitivity and resistance; however, its increased expression is detrimental [97,98,99,100]. Evidence has been given of mice with neuronal SOCS3 or PTP1B deficiency that have improved sensitivity to leptin and insulin, and are resistant to increases in body weight when exposed to high-fat diets [92]. Animal models with suppression or precise variations of SOSC3 have shown greater sensitivity to leptin, in addition to showing resistance to DIO, weight loss, and decreased food intake [68]. In a study using risperidone in the human neuroblastoma cell line (SH-SY5Y), the authors found increases in both SOSC3 and suppressor of cytokine signalling type 6 (SOCS6) proteins through the cyclic adenosine monophosphate/protein kinase A/ERK pathway (cAMP/PKA/ERK); the drug causes excessive weight gain due to inhibition of leptin and insulin signalling pathways [101]. In a fasting-induced hyperphagia assay, mice lacking the SOCS3 protein in LepR-expressing cells (LepR SOCS3KO) showed increased leptin sensitivity in the hypothalamus, and hyperleptinemia was also prevented [98]. Furthermore, one paper finds that leucine supplementation improves leptin sensitivity in HFD rats by promoting leptin signalling and downregulating SOCS3 expression [97].

An interesting assay involving the orexigenic hormone ghrelin shows a significant increase in exchange protein activated by cAMP (Epac) and SOCS3 in cultured rat nodose ganglia (NG) neurons that significantly inhibits the anorexigenic hormone leptin-induced STAT3 phosphorylation. From the study, the authors conclude that the SOCS3 signalling pathway plays a fundamental role in the inhibitory effect of ghrelin on leptin-induced STAT3 phosphorylation [100]. Other works conclude that the deregulation of SOCS3 expression and activity may be involved in leptin resistance by interrupting the negative feedback loop [70,99]. However, Pedroso et al. [99] indicate in their report that the mechanism involved in the inhibition of SOCS3 in LepRb-expressing cells could depend on specific neurons, finding that it cannot prevent diet-induced obesity. Therefore, we conclude that the alteration of the negative feedback pathway SOCS3 could somehow be involved in leptin resistance.

Specific inhibitors for SOCS3 have not been developed [70], although they may be an option to restore the leptin response. A limitation in their development is the need to act specifically on the neuronal circuits involved in the regulation of body weight since its inhibition can cause serious alterations. Some suggested pharmacological strategies for SOCS3 inhibition are microRNA therapy and administration of zoledronic acid [102]. Inhibition has also been suggested with a specific strategy at the level of the leptin receptor pathway [70]. Furthermore, following a study in which it was discovered that the inhibition of the cell cycle checkpoint protein Ataxia Telangiectasia and RAD3-related protein (ATR) leads to a decrease in the expression of SOCS3, the use of ATR inhibitors in the development of new treatments for obesity may be useful [103].

Additionally, we will review some works that implicate the increased expression of the negative regulatory molecules PTP1B and TCPTP in leptin resistance. Thus, PTP1B has been linked to central leptin resistance in humans and in a variety of animal models of obesity and aging [4]. Similarly, in a study using the intranasal route of administration, suppression of PTP1B/TCPTP in the ARC was shown to restore downstream leptin responses. They also suggest that the combined deletion of PTP1B and TCPTP in the ARC of obese mice may produce synergistic effects that contribute to decreased food intake and weight loss. Conversely, in the aforementioned report, it is shown that the removal of SOCS-3 did not have an additional impact on body weight [104]. In another study, the authors found that hypothalamic suppression of PTP1B and LepRb failed to rescue hyperphagia or obesity in mice deficient in PTP1B and LepRb [Nkx2.1-LepRb(-/-)]. Thus, they suggest that functional leptin receptor signalling is in fact required for the metabolic effects of PTP1B. Likewise, they propose two models: the first where PTP1B deficiency exerts beneficial effects because of increased sensitivity to leptin, and a second model in which the consequences of PTP1B deficiency are achieved through the combination of different signalling pathways, where leptin plays a fundamental role [105]. Furthermore, we can suggest that additional signalling pathways in the brain may contribute to the metabolic effects produced by inhibiting PTP1B. Thus, another study shows that PTP1B overexpression reduces phosphorylation of the tropomyosin receptor kinase B (TrkB) receptor and the activation of downstream signalling pathways, while PTP1B inhibition increases TrkB signalling. Considering that TrKB implicates PTP1B in the regulation of central brain-derived neurotrophic factor (BDNF) signalling, the study suggested that the beneficial effects of PTP1B inhibition would be exerted by enhancing BDNF/TrkB signalling [106].

Yet another work studies the effects of Roux-en-Y gastric bypass (RYGB) in relation to the hypothalamic suppression of PTP1B. The results obtained therein show that leptin levels were elevated in obese rats and decreased after RYGB. In addition, the expression of the anorexigenic peptide POMC increased and that of the orexigenic peptide NPY decreased in RYGB rats. Furthermore, RYGB surgery decreases PTP1B expression and improves leptin sensitivity through its effects on the leptin signalling cascade [107]. The importance of this work must be highlighted since RYGB surgery is an effective treatment for obesity and the fact that it decreases the expression of PTP1B and produces an improvement in leptin sensitivity provides further support to the hypothesis. In another study, improvements in leptin and ghrelin levels were found after bariatric surgery which may involve multiple mechanisms, such as a reduction in inflammation and better glycemic control [108].

Elsewhere, research linking leptin to DIO has reported cases in which the elevated hypothalamic expression of PTP1B and TCPTP has been implicated in leptin resistance. Evidence on the subject has found that in POMC neurons, overexpression of the unfolded protein response transcription factor (Xbp1s) decreases the expression of PTP1B and SOCS3, improves leptin and insulin sensitivity, and protects against obesity. Furthermore, the combined deletion of PTP1B and TCPTP in neuronal and glial cells or POMC neurons results in an improvement in leptin sensitivity as well as a decrease in diet-induced obesity [109].

The evidence reviewed allows us to affirm that the inhibition of PTP1B and TCPTP positively affects resistance to hypothalamic leptin induced by different causes. Thus, academic and pharmaceutical research has pointed to PTP markers as therapeutic targets and has been implicated in the development of PTP1B and TCPTP inhibitors. Although the development of PTP1B and TCPTP inhibitors that target specific regions of the brain is a major challenge, current technological advances make it feasible. Thus, future research should seek a chemical molecule with better pharmacological properties and fewer side effects [109,110,111]. However, for an expansion on the subject and to learn about the variety of inhibitors developed, there are previous reviews [109,110]. Thus, a study that synthesised the compound ethyl-3-(hydroxymethyl)-4-oxo-1,4-dihydrocinnoline-6-carboxylate (PI-04) (a PTP1B inhibitor), shows that it improves the stimulating effect of leptin on the phosphorylation of the insulin receptor substrate 2 (IRS2) and of the transcription factor STAT3 [112]. Similarly, in another report using DIO mice, JTT-551, a novel inhibitor of PTP1B, is evaluated. The results of the study confirm an inhibitor-induced anti-obesity effect that enhanced leptin signalling [113]. Similarly, in a study evaluating the anti-diabetic and anti-obesity impact of the inhibitor 4-(biphenyl-4-ylmethylsulfanylmethyl)-N-(hexane-1-sulfonyl) benzoylamide (KY-226), it was found that oral administration of KY-226 decreased weight gain, food consumption, and fat volume gain, in addition to finding increases in phosphorylated STAT3 in the hypothalamus [114].

From what has been discussed so far, it can be concluded that the inhibition of PTP1B and TCPTP in the ARC, and even in other brain areas, could be effective in improving cellular leptin resistance. Furthermore, whether the effects of PTP1B/TCTPP inhibition in the hypothalamus are sufficient to increase leptin sensitivity independently or whether other signalling pathways are involved merits further investigation. On the other hand, it would be reasonable to focus research on the search for PTP inhibitors since there are more possibilities of obtaining an effective inhibitor of PTP1B than an inhibitor of SOSC3. In addition, as already pointed out, the deletion of PTP1B/TCPTP and SOCS3 does not produce synergy and actually, as a study of this literature shows, the elimination of SOCS3 does not imply an additional impact on the beneficial results obtained with the deletion of PTP1B and TCPTP. However, the research involving the SOCS3 pathway should not be abandoned, the implications of each protein in leptin resistance must be delimited.

It has been proven that leptin receptor signalling pathways may be affected by different mechanisms to produce leptin resistance. In addition, more than one signalling pathway may be affected and their detrimental effects may be synergistic or interrelated. In turn, the discovery of new pathways or inhibitory mechanisms could bring about new advances in the field.

In a recent study, a signalling pathway called NSAPP (NADPH oxidase-4, Superoxide dismutase, Aquaporin-3, PTEN) was found that could provide new information since it regulates the expression of neuropeptides such as POMC and agouti-related peptide (AgRP) which control appetite. Furthermore, it has been suggested that a defect in this pathway could explain the simultaneous resistance to the appetite-suppressing effects of leptin and insulin in obesity. Interference with this pathway in lean animals is also hypothesised to lead to overeating, positive caloric imbalance, and weight gain [58]. Similarly, additional research identifies a molecular signalling pathway linking the gastric inhibitory polypeptide receptor (GIPR) with overnutrition via EPAC/Ras-related protein 1 (Rap1) in the brain. Its authors found that central administration of gastric inhibitory polypeptide (GIP) decreased hypothalamic leptin sensitivity and increased hypothalamic SOCS3 levels, and GIPR deficiency protected against diet-induced leptin resistance [115].

Interestingly, in a study that administered pituitary adenylate cyclase-activating polypeptide (PACAP) to the ventromedial nucleus of the hypothalamus (VMN), it was observed that increased STAT3 phosphorylation and SOCS3 mRNA expression, effects that mimic those of leptin receptor activation, took place. Furthermore, BDNF mRNA expression in the VMN was increased by both leptin and PACAP administration, and a PACAP receptor antagonist reversed the effects of leptin in the VMN. Consequently, the authors suggest a common signalling pathway for both polypeptides [116]. The inducible inhibitor hexamethylene bis-acetamide inducible-1 (Hexim1) has also been shown to regulate the expression of transcription factors (SOCS3 and STAT3) in the hypothalamus, skeletal muscle, and adipocytes. These factors modulate obesity, and it is suggested that Hexim1 could play a central role in maintaining the energy balance of the whole body [117]. Other research suggests that the cAMP/PKA pathway plays an important physiological role in the modulation of LepRb signalling gain in the hypothalamus and this could lead to changes in adiposity. Thus, for the authors of the study, these results represent an opportunity to investigate new therapeutic strategies to increase leptin sensitivity through the cAMP and LepRb signalling pathways [118]. Finally, a recent study reports dysfunction in the Akt and phosphodiesterase 3 B (PDE3B)/cAMP pathways of leptin signalling in the hypothalamus that contribute to the development of central leptin resistance and DIO [119].

In summary, the decrease in the satiety signal of leptin in leptin resistance can occur due to the different mechanisms described or it may be caused by the ineffective result of a combination of these mechanisms.

## 6. Leptin Signalling, Obesity, and Alzheimer’s Disease

There is a rare and genetically established form of Alzheimer’s disease (AD) and a sporadic form which is the most common in Alzheimer’s patients. In turn, there are genetic variants associated with an increased risk for Alzheimer’s disease. The best known is a variant of the gene encoding apolipoprotein E (APOE), the variant being APOE4 [120]. Furthermore, the disease is associated with the formation of extracellular β-amyloid (Aβ) plaques and the accumulation of hyperphosphorylated tau proteins in the brain. Mitochondrial dysfunction is a key process underlying AD pathology, where mitophagy (or selective degradation of mitochondria by autophagy) and autophagic pathways seem to be altered in this pathology [121]. Indeed, these processes, when inadequately regulated, contribute to synaptic dysfunctions and cognitive impairments through the accumulation of Aβ fibrils and hyperphosphorylated tau protein tangles. Moreover, the transcription factor EB (TFEB) has been described as a molecular target to treat neurodegenerative disorders such as AD, Parkinson’s disease, or Huntington’s disease. TFEB is a principal regulator of autophagy and lysosomal biogenesis pathways [122], the malfunction of which leads to neuronal death. Moreover, phytoconstituents have been proposed as potential therapeutic remedies to manage AD [123]. The same work indicates the use of nanocarrier systems, whose use led to the correct delivery of herbal medicaments to a specific target, although the use of phytoconstituents is limited due to their low solubility and metabolism. Additionally, compounds have been developed with therapeutic applications for the symptomatic effects of AD, such as oxidative stress and learning and memory [124,125]. These are the piperazine derivative biphenyl-3-oxo-1,2,4-triazine and benzoxazole derivatives which seem to act as cholinesterase inhibitors and have antioxidant properties, enhancing learning and memory. Similarly, in AD, there is not only synaptic dysfunction but also neuroinflammation and oxidative damage that affects episodic memory and leads to impaired cognitive functions which cause interference in patients’ lives [126,127]. Obesity is also a risk factor for dementia and Alzheimer’s disease.

Given the relationship between obesity and dementia, some scientists are beginning to investigate a link with leptin. AD has been considered as a brain-type metabolic disorder [126,128,129] which requires a readjustment of homeostasisq and is influenced by inflammation, adipokines, adipocyte-derived hormones, and the various exposed mechanisms of leptin resistance. Thus, neuronal resistance to leptin is suggested in AD [130,131].

The link between obesity, leptin, and AD is now well established and has been extensively studied. Thus, different epidemiological studies have found an association between AD and changes in body weight. Some reports have found that obesity in midlife, as well as weight loss in old age, are related to cognitive decline and increased risk of developing AD [62,132,133,134]. Diets rich in saturated fatty acids have also been associated with an increased risk of AD in several animal and human studies [133] and several studies have found an association between a low body mass index (BMI) and AD in post-mortem brains [62,133]. Furthermore, it has also been suggested that WHR, but not BMI, was correlated with an increased risk of AD [132]. However, a study from the United Kingdom shows that the chance of developing AD is lower in obese middle-aged people but higher in middle-aged and older people of low weight [62,133]. In summary, although there is no unanimity across the studies, most of the evidence suggests that the increase in adiposity during middle age influences the risk of developing dementia, as well as its reduction in old age. So, as the disease develops before the appearance of cognitive symptoms, it is suggested that low weight in old age may be a manifestation of this early stage of the disease and a sign of premature brain dysfunction [62,133]. In support of this notion, low BMI has been found to be associated with the deterioration of AD pathology in postmortem brains, as well as the worsening of CSF biomarkers {tau and amyloid-β peptide form (Aβ1-42)} [62,133]. Interestingly, some models have been proposed to explain weight change at different stages of AD [62,133].

It has been shown that leptin is secreted by adipocytes and circulates in plasma in proportion to fat mass [48,63,130], and changes in body weight are associated with the possibility of developing AD. Therefore, it is not surprising that different investigations try to relate dysfunctional levels in leptin signalling with AD. Thus, in some studies, low plasma leptin levels in old age have been found to be associated with an increased risk of cognitive decline and AD development [62,134]. Similarly, the authors of one study show that plasma leptin levels are lower in subjects with mild cognitive impairment (MCI) or AD compared to control subjects and confirm a decrease in Aß42 and an increase in the tau protein related to MCI and AD. In addition, the same study suggests that plasma leptin deficiency could indicate a possible CNS leptin deficiency and thus serve as a diagnostic marker for MCI or AD [135]. Several studies also reported the same evidence in subjects with MCI or AD [4,62,136].

Not all studies have found an association between circulating leptin levels and AD or cognitive decline; one study finds no relationship between leptin levels and cognition, or disease severity in AD patients. Moreover, unlike other previous investigations that found positive correlations between leptin levels, BMI, body weight, and mean waist circumference, they also found no correlation between leptin levels and the aforementioned measures [137]. Similarly, another recent report finds that serum leptin levels were similar in young patients (mean 60 years) diagnosed with AD and vascular dementia, compared to healthy controls and patients with subjective memory complaints, and concluded that peripheral leptin levels do not play a role in the evolution of AD pathology [138]. Similarly, another current study reported no significant change in CSF leptin levels in AD compared to controls, at least in the early phases of AD progression during which the BBB remains intact [130]. These contradictory aspects have been explained by methodological differences or deficiencies and the complexity of leptin itself, although a non-linear U-shaped relationship has also been proposed, as we mentioned previously [64].

On the other hand, evidence suggests that leptin is involved in memory impairments that occur in hippocampal-dependent AD. Thus, leptin treatment improves performance on hippocampal-dependent memory tasks in SAMP8 mice that have elevated Aβ levels [66] and prevents the detrimental effects of Aβ on LTP and LTD in the hippocampus, as well as on AMPA receptor trafficking, and inhibits Aβ-induced up-regulation of endophilin 1 expression. In addition, there is evidence indicating that toxic Aβ levels decrease with leptin treatment. Thus, the authors suggest a dysfunction of the leptin signalling cascade involved in Aβ actions at hippocampal synapses [59,66]. In view of this evidence, a relationship between leptin and AD can be assumed in which very high levels of leptin, probably due to leptin resistance, as well as low leptin levels are associated with a higher risk of AD.

It seems that the leptin detected in the brain comes from the periphery [130]. Therefore, analysing the transport of leptin through the BBB and its integrity is essential to understanding the levels of leptin and its signalling in the brain. Although the BBB has been shown to be compromised in AD brains, it appears that such impairment may occur late in the disease [130]. Thus, a study that analysed whether leptin levels change as the subject progresses towards AD found no differences in these leptin levels in the CSF in the early stages of AD progression, nor did it detect changes in the CSF albumin/serum albumin ratio, reflecting the integrity of the BBB. For the authors of the study, these results suggest that leptin levels are intact, but leptin signalling is altered in AD [130]. We can conclude that the integrity of the BBB may be affected in the advanced phase of the disease and there seems to be a serious disconnection in the leptin signalling pathway in AD. In fact, a central resistance to leptin is mentioned in AD by detecting reductions in some signalling pathways that are activated downstream of leptin receptors [130].

It has been detected that the expression of the leptin receptor is altered in AD. Thus, in one study, it was found that the expression level of LepRb mRNA was lower in hippocampal tissue with AD compared to controls. Similarly, this study suggests a possible blockade induced by neurofibrillary tangles (NFT) to the accessibility of LepRb; thus, the leptin receptor is sequestered in regions with high concentrations of NFT which causes it to lose its signalling capacity. The lack of signalling results in the interpretation that there are inadequate circulating leptin levels, leading to increased leptin secretion and leptin resistance in these affected neurons [131]. Similarly, in another study with young and old mutant mice expressing APPSWE (Tg2576), a reduction in leptin was detected in neurons compared to those of control animals and young Tg2576 mice have also been found to have enhanced expression of LepRb, which is not seen in old Tg2576 mice. Therefore, a dysfunction in leptin signalling is suggested [130].

We might say that age is an important risk factor for dementia and specifically for AD. Similarly, diet, lifestyle, and stress also influence the development of dementia and low weight in old age and obesity in middle age are predisposing factors. Furthermore, the data analysed suggest that obesity in midlife and the elevated leptin levels seen in the obese lead to leptin resistance in the brain, which also predisposes to AD. Subsequently, before the manifestations of the disease, obesity-induced systemic inflammation will lead to neurodegeneration, which increases with age, where leptin signalling pathways are affected, altering their function.

Therefore, inflammation is involved in neurodegeneration, and it is present in the pathophysiology of AD. The peptide β-amyloid (Aβ) has been observed to influence the function of adipose tissue. Thus, it has been found that the fragment of the β-amyloid peptide (Aβ25-35) through protein kinase A (PKA) and ERK 1/2 dependent signalling pathways, can cause an increased release of free fatty acids and proinflammatory adipokines that can produce lipotoxicity in other organs [139]. Likewise, a high sucrose diet (HSD) fed to male APPswe/PS1dE9 (APP/PS1) transgenic mice induced an increase in neuroinflammation, as well as an increase in cortical and serum levels of β-amyloid. This work demonstrates that a HSD increases AD-related pathologies and reduces hypothalamic leptin signalling in APP/PS1 mice [140]. Similarly, a study investigating amyloid β-secretase (BACE1)-dependent processing in relation to leptin resistance demonstrates the need for functional leptin signalling and shows that reducing BACE1 activity is found to restore leptin sensitivity, normalise hypothalamic inflammation, and reduce PTP1B and SOCS3 [141].

However, it seems that Aβ and tau cannot fully explain the pathology of AD. Thus, a study has found a decrease in STAT5B and SOCS(1-3) in the brains of transgenic mice (APP/PS1) at 3 months of age. These results demonstrate a significant impairment in adipokine receptor signalling pathways in the hippocampus of APP/PS1 mice at a young age, before Aβ plaque formation [128]. Similarly, in another study using high-fat diet (HFD)-treated (APP/PS1) transgenic mice, leptin failed to suppress the food intake of HFD APP/PS1 transgenic mice, suggesting impaired signalling of leptin in the hypothalamus, but hyperphagia and aggravated obesity were caused before the presence of amyloid pathology [142].

As mentioned in the previous section, protein tyrosine phosphatase 1B (PTP1B) is another mechanism involved in leptin resistance and works by modulating signalling pathways related to learning and memory, endoplasmic reticulum (ER) stress, and microglia-mediated neuroinflammation [143]. Therefore, the effects of its inhibition merit further investigation.

On the other hand, leptin, through its signalling pathways, can modify beta-amyloid peptide (Aβ) levels by blocking β-secretase activity and increasing ApoE-dependent amyloid beta uptake; it can also enhance Aβ peptide clearance by promoting its clearance and degradation [137]. Furthermore, leptin inactivates glycogen synthase kinase 3 β (GSK3β), the protein primarily responsible for tau hyperphosphorylation [127,132]. Adenosine monophosphate protein kinase (AMPK)/sirtuin1(SIRT1)-activated pathways appear to mediate the reduction of β-secretase activity by leptin, and AMPK, Akt protein, and p38 protein pathways mediate the reduction of tau phosphorylation by leptin [62].

**Figure 1:**
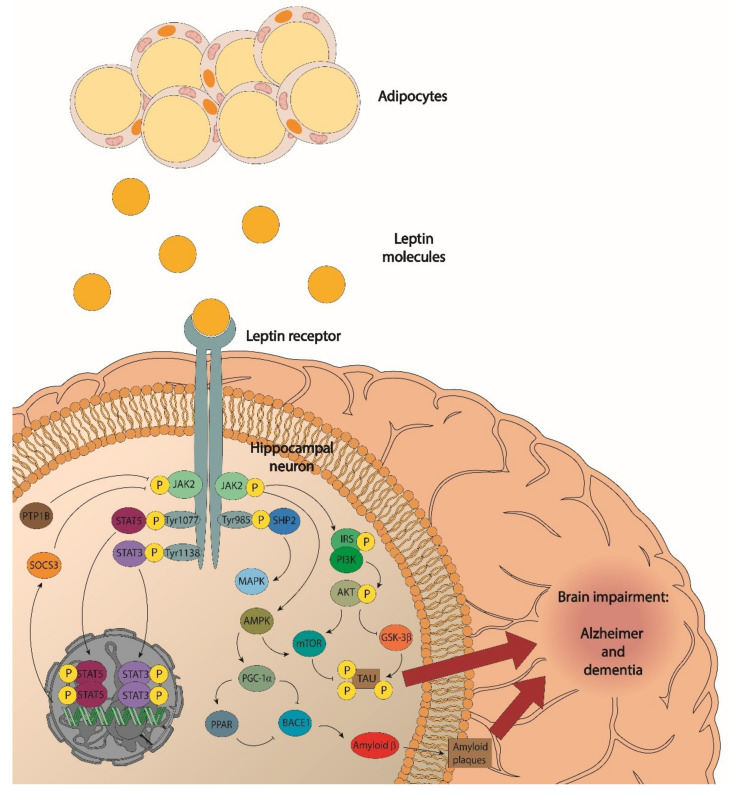
Leptin signalling in a hippocampal neuron. Leptin, mainly released by adipose tissue, reaches a hippocampal neuron where, binding to LepRb, activates the JAK/STAT, PI3K/Akt, and AMPK signalling pathways. PI3K/Akt pathway activation leads to mTOR activation, influencing synthesis and aggregation of Tau, as well as GSK-3β inactivation, which, in turn, is not able to hyperphosphorylate Tau. AMPK pathway activation leads to PGC-1α and PPAR activation, which, translocated to the nucleus, inhibit BACE1 transcription and decrease Aβ production. SOCS3 and PTP1B are negative regulators of leptin signalling acting on JAK2 and, therefore, generate leptin resistance that may cause the worsening of AD.

So, LepRb, expressed in the hippocampus, triggers leptin signalling via Janus Kinase 2 (JAK2), which, in turn, phosphorylates three tyrosine residues in the LepRb intracellular domain (Tyr985, Tyr1077, and Tyr1138). Tyr1138 mediates the activation of STAT3 [144]. STAT3 is a transcription factor that, phosphorylated by JAK2, dimerises, and is translocated to the nucleus, where it controls the transcription of specific genes, within which is the suppressor of cytokine signalling 3 (SOCS3), a negative regulator of LepRb intracellular signalling [145,146]. The phosphorylation in Tyr1077 induces STAT5 activation [147]. Phosphorylated Tyr985 recruits the SH2-containing tyrosine phosphatase-2 (SHP-2) to mediate the activation of the MAPK pathway (extracellular signal-regulated kinase or ERK) [144,148]. Activated JAK2 can initiate the PI3K/Akt signalling pathway through the phosphorylation of IRS protein [144] which leads to the mammalian target of rapamycin (mTOR) activation, which influences the synthesis and aggregation of Tau, compromising microtubule stability when mTOR is inactivated [149]. Moreover, the PI3K/Akt signalling pathway can also inactivate glycogen synthase kinase 3 β (GSK3β), which, in turn, is unable to phosphorylate the Tau protein [150,151], thereby unfavouring the Tau aggregation characteristic of AD. Another key downstream effector of LepRb includes AMP-activated protein kinase (AMPK), which is phosphorylated by JAK2 [152]. AMPK may, in turn, induce a decrease in tau phosphorylation and β-amyloid accumulation [153]. These effects are PGC-1α-mediated, which is a coactivator of peroxisome proliferator-activating receptor γ (PPARγ), a transcriptional activator of target genes, regulating the transcription of BACE1, which is reciprocally regulated with respect to PGC-1α expression [154]. BACE1 is a key enzyme involved in Aβ generation, the hallmark harmful peptide of AD. A reduction in the activity and expression levels of BACE1, with a decreased production of Aβ after leptin stimulation, has been described [155]. Conversely, leptin signalling can be reduced by contra-regulatory mediators, such as SOCS3 and protein tyrosine phosphatase 1B (PTP1B). After leptin binding to its LepRb receptor, SOCS3 interferes with LepRb/JAK2 signalling [145], whereas PTP1B has the ability to dephosphorylate JAK2 [156]. Research has shown that mRNA, SOCS3, and PTP1B, are up-regulated in the hippocampus of an animal model of AD [157], suggesting that they may generate leptin resistance. (Figure 1).

In summary, it can be said that LepRb signalling pathways seem to be clearly involved in AD. We can postulate that obesity in midlife increases the risk of AD by promoting systemic inflammation and leptin resistance, which leads to brain neurodegeneration. Subsequently, neurodegeneration worsens, creating a vicious cycle that leads to AD pathology where inflammation and oxidative stress become important factors in its development and progression [126].

## 7. Conclusions

Because obesity is a risk factor for AD and other dementias [4,6,50,52], effective markers should be determined in order to prevent adverse effects, as well as begin comprehensive programs for prevention. Indeed, AD and obesity share common metabolic characteristics. Thus, adipokines, secreted by adipose tissue, communicate with the CNS, playing a role in the progression of AD and other dementias [6]; among them, leptin [4] has neuroprotective effects at the level of CNS [63,65]. Properly identifying the alterations in the signalling pathways triggered by leptin and its receptor, LepRb, is necessary in AD in order to reverse the dysfunction of these pathways and consequently improve the prognosis of AD. In fact, different mechanisms of leptin resistance have been described, among which are those carried out by SOCS3, PTP1B, and TCPTP, where SOCS3 and PTP1B have an altered expression level in an animal model of AD [157], disrupting leptin signalling. Thus, research should be aimed at the design of inhibitors or related mechanisms to reduce this resistance to leptin. In fact, the design of inhibitors against protein tyrosine phosphatases is possible [104], and the use of ATR inhibitors to reduce SOCS3 expression has also been suggested [103]. CNS inflammation or neuroinflammation is an important factor in the development and progression of neurodegenerative diseases [29,30,31], such as AD, PD, or HD, and inflammation can affect leptin-triggered signalling, leading to leptin resistance [158]. Thus, the use of anti-inflammatory drugs combined with those that reduce leptin resistance could be useful.

## Data Availability

Not applicable.
